# Effects of a Seven Day Overload-Period of High-Intensity Training on Performance and Physiology of Competitive Cyclists

**DOI:** 10.1371/journal.pone.0115308

**Published:** 2014-12-18

**Authors:** Bradley Clark, Vitor P. Costa, Brendan J. O'Brien, Luiz G. Guglielmo, Carl D. Paton

**Affiliations:** 1 School of Health Science, Federation University, Ballarat, Victoria, Australia; 2 Physical Effort Laboratory, Federal University of Santa Catarina, Florianópolis, Brazil; 3 Faculty of Health and Sport Science, The Eastern Institute of Technology, Hawkes Bay, New Zealand; University of Buenos Aires, Faculty of Medicine. Cardiovascular Pathophysiology Institute, Argentina

## Abstract

**Objectives:**

Competitive endurance athletes commonly undertake periods of overload training in the weeks prior to major competitions. This investigation examined the effects of two seven-day high-intensity overload training regimes (HIT) on performance and physiological characteristics of competitive cyclists.

**Design:**

The study was a matched groups, controlled trial.

**Methods:**

Twenty-eight male cyclists (mean ± SD, Age: 33±10 years, Mass 74±7 kg, VO_2_ peak 4.7±0.5 L·min^−1^) were assigned to a control group or one of two training groups for seven consecutive days of HIT. Before and after training cyclists completed an ergometer based incremental exercise test and a 20-km time-trial. The HIT sessions were ∼120 minutes in duration and consisted of matched volumes of 5, 10 and 20 second (short) or 15, 30 and 45 second (long) maximal intensity efforts.

**Results:**

Both the short and long HIT regimes led to significant (p<0.05) gains in time trial performance compared to the control group. Relative to the control group, the mean changes (±90% confidence limits) in time-trial power were 8.2%±3.8% and 10.4%±4.3% for the short and long HIT regimes respectively; corresponding increases in peak power in the incremental test were 5.5%±2.7% and 9.5%±2.5%. Both HIT (short vs long) interventions led to non-significant (p>0.05) increases (mean ± SD) in VO_2_ peak (2.3%±4.7% vs 3.5%±6.2%), lactate threshold power (3.6%±3.5% vs 2.9%±5.3%) and gross efficiency (3.2%±2.4% vs 5.1%±3.9%) with only small differences between HIT regimes.

**Conclusions:**

Seven days of overload HIT induces substantial enhancements in time-trial performance despite non-significant increases in physiological measures with competitive cyclists.

## Introduction

For many endurance athletes training is periodised across the season in order to prepare for competitions. The structure of training within a specific phase of a season often varies, and can include a combination of training techniques which are ultimately designed to enhance the athlete's performance capacity through increases in maximum oxygen consumption (VO_2_ max), the sustainable percentage of maximum oxygen consumption (often erroneously referred to as anaerobic threshold) or aerobic economy/efficiency. Whilst low intensity, high volume training plays a major role in an endurance athlete's preparation there is little doubt that bouts of higher intensity training (HIT) are necessary in order to enhance athletic form and particularly VO_2_ max [1]. Further a common practice observed amongst competitive athletes is to include short periods of heavily intensified training (often in the form of overload HIT or minor competitions) immediately prior to important competitions in order to further enhance race performance.

The structure of HIT sessions are diverse but generally involve short (<5 minutes) repeated bouts of maximal intensity exercise at or above an athlete's maximum oxygen consumption power [2]. In recent years there have been a number of studies investigating the effects of various specific HIT regimes on an athletes' performance and physiological characteristics. In an early study Stepto et al., [3] examined the effects of six sessions (completed over two weeks) of different varieties of HIT programmes on 40-km time-trial performance with well-trained cyclists. Interestingly this study reported that the largest improvements in time-trial performance occurred from two quite diverse (30-s vs 240-s) HIT programmes, unfortunately this study did not examine the physiological mechanisms underlying any of the observed performance enhancements from the HIT sessions. Similarly in a series of related HIT studies with competitive cyclists, Laursen and colleagues [4]–[6] reported significant improvements in time-trial performance following 2–4 weeks of different duration (>30-s) maximal intensity intervals. The improvements in performance, in this series of studies, were associated with a significant increase in two of the recognised determinates of endurance performance, namely maximal oxygen consumption and lactate threshold power. Combining short duration (30-s) sport specific HIT with non-specific explosive training has also been shown to substantially enhance the third physiological determinant associated with endurance performance, namely aerobic economy, in both cyclists [7] and runners [8].

As it is apparent that quite diverse forms and durations of HIT are an effective training strategy [2], more recent research has focused on the organisation and distribution of the HIT sessions within a periodised training program. In a study examining the effects of three weeks (nine sessions) of HIT performed on either consecutive or non-consecutive days, Gross et al., [9] reported similar improvements in performance and physiological measures following either strategy with recreational level cyclists. Furthermore two other recent studies indicate performance may be enhanced if a short block of concentrated interval training is followed by a three week period of less frequent interval training [10], [11].

Regardless of its configuration, training is often organised to induce a state of temporary but functional overreaching. Functional overreaching is a training state that results in a short term performance decrement that, when followed by an adequate period of recovery, results in super-compensation and subsequent performance enhancement [12]. To evoke short term functional overreaching coaches and athletes often include short periods of highly intensified training, such as a training camp or low priority competition, in the weeks preceding a major competition. Previous research has established the potential for a very short period of intensified block training, such as would occur during a training camp or race, to improve performance. Jeukendrup et al. [13], reported significant improvements in performance after competitive cyclists had undertaken two weeks of recovery following a two-week period of intensified training. In a similar study investigating the effects of induced overreaching, Halson et al., [14] found brief periods of highly intensified training can lead to a decline in performance that may be sustained for periods of up to two weeks following the training period. However, this study did not include any longer term monitoring so it is unknown if any super-compensation effects occurred after the two week recovery period. Consequently it appears from observation and previous research that approximately seven days of intensified training maybe the optimal duration for improving performance without causing undue long term fatigue. Therefore while it appears different forms of HIT can lead to substantial performance gains, further research is warranted to determine the effects of shorter block periods of intensified training on the physiology of trained cyclists and the time course of any performance enhancements. To our knowledge no previous study has examined the magnitude of performance gains possible following a typical seven day intensified training period. Therefore the aim of this study was to determine the effects of seven consecutive days of two different HIT programs which simulated the intensity of efforts seen in competition, on the physiological and performance adaptations of competitive cyclists, and also to examine the time course of any adaptations during the post-training recovery period.

## Methods

### Subjects

Thirty competitive male cyclists initially volunteered to participate in this study. Two cyclists failed to complete all sessions due to illness unrelated to the study and were therefore excluded from the final analysis leaving a total of 28 cyclists (Mean ± SD, age: 33±10 years, mass 74±7 kg, height 178±5 cm, VO_2_ peak 4.7±0.5 L·min^−1^) at completion. All cyclists gave their written informed consent to participate in the study which was prior approved by the participating Universities human research ethics committees in accordance with the declaration of Helsinki. The cyclists were well-trained with a minimum of two years competitive experience at grade A or B (Oceania amateur grading). The study was performed in the competitive season following a period of base and pre-competition training. Due to the nature of each cyclist's competition programme it was not possible to control their individual training leading up to the study. However immediately prior (2 weeks) to the start of the study cyclists were completing individual self or coach-determined training regimes consisting of a minimum of ten hours (∼300-km) mixed intensity training per week. At the start of laboratory testing cyclists were required to be in a well-prepared and non-fatigued state.

### Testing procedures

**Figure 1:**
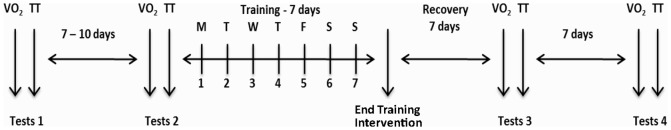
Sequence of training and testing followed by the cyclists in the experimental groups; control group subjects completed tests 2 and 4 only. VO_2_  =  Maximal incremental exercise test, TT  =  time trial.

Cyclists were matched as closely as possible based on peak power output and peak oxygen consumption (VO_2_ peak) from the initial incremental test, and assigned to one of three conditions; a control group (n = 9), short sprint (n = 9) and long sprint (n = 10) HIT groups. The control group completed two physiological and performance assessments separated by three weeks during which they continued with their normal prescribed training (minimum of ten hours per week). The cyclists in the training groups completed a series of physiological and performance assessments before and after completing a seven day block of intensified training. The sequence of testing and training for all subject groups is shown in Fig. 1. All physiological and performance assessments were completed on a Velotron Dynafit Pro cycle ergometer (RacerMate Inc, WA, USA) using the company's associated 3D race and coaching software. Prior to testing each participant was fitted to the ergometer in a position to replicate their own racing bicycle; the fit measurements were recorded and repeated for each subsequent session. In the 24 hours before any testing session, participants were instructed to prepare as if it was a competition, and to avoid strenuous physical activity and any potential performance altering supplements (e.g. caffeine). Participants reported to the laboratory approximately 30-minutes prior to each test having slept a minimum of seven hours and in a well fed and hydrated state. Throughout all tests, cooling was provided via two 30 cm pedestal fans and the ambient temperature of the laboratory was controlled at ∼20°C with a relative humidity of ∼50–60%.

### Incremental exercise test

The physiological assessment consisted of an incremental exercise test to volitional exhaustion, from which measures of peak power output (PPO), VO_2_ peak, power at the 4 mmol/L lactate point (OBLA), aerobic economy and efficiency were assessed. During the incremental exercise test respiratory gases were continuously measured with a metabolic cart (Metalyser 3B, Cortex, Leipzig, Germany) calibrated in accordance with the manufacturer instruction using Alpha gas standards. Cyclists initially began exercising at 100 watts (W) increasing by 40 W every four minutes thereafter until reaching volitional exhaustion. The ergometer was set to isokinetic mode during the incremental test so that power output remained constant regardless of changes in pedal cadence. Cyclists were allowed to freely vary there cadence during the test though were encouraged to maintain a cadence of ∼90 revolutions per minute. During the final 30 seconds of each stage 25 µL of blood was collected from the participant's fingertip and immediately analysed for whole blood lactate concentration using an automated system (YSI 1500, Yellow Springs, OH, USA) calibrated to the manufacturer's specifications. Peak power output in the incremental test was determined as the final completed stage plus the proportion of any uncompleted stage reached during the graded exercise test in accordance with Lucia et al., [15]. Peak oxygen uptake was determined as the highest 30 second oxygen uptake value recorded during the test. The onset of blood lactate accumulation (OBLA) was determined as the power output at which blood lactate reached a concentration of 4 mmol/L. Aerobic economy was determined as the oxygen consumption at a fixed power of 220 W for all subjects as this was the highest intensity achieved in all subjects where oxygen consumption remained at steady state and the respiratory quotient was <1.0; similarly gross efficiency (GE) was determined from respiratory data at 220 W in accordance with the methods of Horowitz et al., [16].

### Time trial test

**Figure 2:**
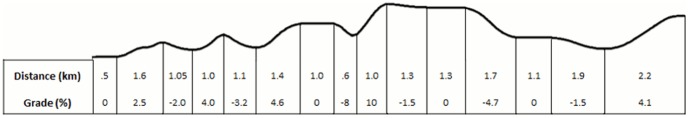
Computer simulated course showing the distances and gradient profile used in the 20-km time trial.

The time trial (TT) was completed on a computer simulated 20-km course using the same ergometer as previously described. The developed course was based upon topography of a local racing circuit and consisted of numerous changes in gradient represented by both ascents and descents (Fig. 2). Studies from our laboratory (In press) indicate a coefficient of variation for this test of ∼1% for time and ∼2% for mean power output. Participants were able to view their progress over the course on a computer monitor and were provided with information on distance completed and gear selected; all other information was blinded to remove any potential pacing effect. Participants were requested to complete each time trial as quickly as possible with no restriction on gear selection, cadence or cycling posture (seated or standing). Participants were not restricted to a set pacing strategy and were not coached on how to best ride the course. Throughout the trial participants were able to consume water *ad libitum.* Performance time (TT TIME) and mean power output (TT PO) recorded from the variable gradient time-trial were the main performance measures in this study.

### Training interventions

Cyclists in the two experimental training groups completed seven consecutive days of HIT. The composition of the training sessions was designed to replicate the intensity and duration of efforts seen in real competition and was determined in conjunction with two elite level coaches, using power data collected from competitive cyclists during racing and on previous competition based performance analysis by Ebert et al., [17]. The training sessions, consisted of multiple sets of self-paced maximal intensity sprints and corresponding recovery periods. The work to rest ratio was matched for both groups at 1∶5 (i.e. a 10-s effort would require a 50-s recovery period) and the total session time was ∼120 minutes including a self-selected 15 minute warm up and cool down period. Cyclists in the short training group completed 25 sets of sprints lasting 5, 10 and 20 seconds (each set) completed in sequence for a total work period of 14.6 minutes and corresponding recovery period of 73 minutes. Cyclists in the long training group completed 10 sets of sprints lasting 15, 30 and 45 seconds for a total work period of 15 minutes and corresponding recovery period of 75 minutes. Cyclists in both groups were asked to complete each effort at the highest possible intensity and in the recovery periods, maintain a work rate of ∼30–40% PPO. All training sessions were controlled using pre-recorded audio signals which indicated the duration of the exercise and recovery periods. Cyclists completed the first, fourth and seventh training session under the supervision of one of the researchers using the laboratory ergometer previously described in order to ensure training compliance. The remaining sessions were performed by the cyclists on their own bicycle either on the road or using a stationary ergometer. In the recovery period post the training intervention cyclists were able to resume light recovery intensity training (<120 mins) but were required to refrain from engaging in high intensity exercise or competitions in the 7 days immediately post HIT. The control group continued with their own personal training programmes for a minimum of 10 hours per week to ensure that total training volume was similar to that of the experimental groups.

### Statistical analysis

Simple descriptive statistics are shown as means ± between-subject standard deviations. Data from the study were analysed using both significance, and magnitude based inferential approaches. Significance based analyses were performed using SPSS statistical software, version 20 for Windows (SPSS Inc, Chigago, IL) with alpha set at 0.05. Initially a one way analysis of variance (ANOVA) was performed on the pre-training measures to determine if there were any significant differences between groups for the dependent variables. A repeated measures ANOVA (factor: training group) was then performed to compare changes between groups over time (pre to 2 week post-test). In order to determine where between group differences occurred pair wise comparisons (Bonferonni corrected) were performed via ANOVA using the pre and post dependent measures for each variable. In the magnitude based approach mean effects of training and their 90% confidence limits were estimated with a made for purpose spreadsheet [18] which utilises the unequal-variances t statistic to perform between group comparisons. Group wise comparisons were computed for change scores between the mean values of the two pre-tests and each of the two post-test in the two training groups and between the single pre and post-tests in the control group. Each subject's change score between trials was expressed as a percent of baseline score via analysis of log-transformed values. Data were log-transformed in order to reduce bias arising from any non-uniformity of error in the data. The spreadsheet also computes chances that the true effects are substantial, when a value for the smallest worthwhile change is entered. We used a value of 1% for the performance power measures, as previous research has shown that this value represents the smallest worthwhile enhancement in power for cyclists competing in time-trial events [19]. To date no research has established how percentage changes in physiological measures would translate directly to percent changes in cycling performance, therefore we interpreted changes in our physiological measures using default standardised effects (the change in mean divided by the between subject standard deviation). The magnitudes of the standardised effects for physiological measures only were interpreted and reported using the established effect thresholds of: 0.2, 0.5, and 0.8 for small, moderate, and large effects respectively in accordance with the recommendations of Cohen [20]. Effect size values <0.2 were deemed trivial differences and considered to be not worthwhile.

## Results

Both HIT groups successfully completed 100% of the prescribed training regime over the allotted 7-day period.

No significant differences (p>0.05) were detected between the training groups for any pre-intervention measures. For PPO, OBLA, TTPO, and TTtime there were significant differences between the training groups over time (p<0.001, p = 0.013, p<0.001 and p<0.001 respectively). Pairwise comparisons for PPO, OBLA TTPO and TTtime revealed significant differences between the control group and short HIT group (p = 0.001, p = 0.024, p = 0.006 and p = 0.06 respectively) and control and long HIT group (p<0.007, p = 0.034, p<0.001 and p<0.001 respectively). With the exception of PPO (p = 0.013) there were no significant differences observed between the short and long HIT groups.

Table 1 shows the mean ± SD results for the performance and physiological measures for each of the groups at baseline (Pre) and following (Post) the training period. The control group experienced trivial to small (ES = 0.15–0.23) decreases in performance variables during the monitoring period whilst both training intervention groups reported moderate (ES = 0.51–0.76) enhancements in performance following the HIT interventions. Further, both HIT groups reported small (ES = 0.24–0.47) increases in VO_2_ peak and power output at OBLA and moderate to large (ES = 0.64–1.02) improvements in aerobic economy and gross efficiency, whilst the experimental controls experienced trivial to small (ES = 0.05–0.34) decrements in most physiological measures (in line with the performance decrease) with the exception of aerobic economy and efficiency.

**Table 1:** The Mean ± SD for all measured variables and the % change between Pre and Post testing for each experimental group, and the effect size for the observed % change.

	Control pre	Control post	% Change (ES)	Short pre	Short post	% Change (ES)	Long pre	Long post	% Change (ES)
**TTPO (W)**	286±38	277±39	−3.3±4.2 (0.23)	279±24	291±19	4.6±4.4 (0.51)	277±26	296±25	6.8±5.8 (0.63)
**TT time (s)**	2290±205	2338±213	1.8±2.2 (0.18)	2299±104	2232±84	−2.9±2.6 (0.59)	2320±135	2216±103	−4.4±3.7 (0.74)
**PPO (W)**	345±36	339±37	−1.7±3.3 (0.15)	341±21	353±19	3.6±3.0 (0.57)	337±27	362±28	7.6±2.3 (0.76)
**VO_2_ peak (L·min^−1^)**	4.6±0.5	4.6±0.5	−0.6±6.3 (0.05)	4.6±0.3	4.7±0.4	2.3±4.8 (0.27)	4.7±0.4	4.9±0.5	3.5±6.2 (0.34)
**OBLA (W)**	292±34	282±37	−3.6±6.4 (0.27)	266±21	276±28	3.6±3.5 (0.47)	298±34	306±34	2.9±5.3 (0.24)
**ECO (W·L^−1^**·**min^−1^)**	72.5±4.0	74.1±4.3	2.2±4.3 (0.34)	71.3±4.5	74.0±3.6	3.9±2.8 (0.64)	71.9±3.3	75.3±3.9	4.6±3.5 (0.84)
**GE (%)**	21.1±1.2	21.4±1.3	1.5±4.3 (0.22)	20.7±1.2	21.3±1.0	3.2±2.4 (0.53)	20.8±0.9	21.8±1.1	5.1±3.9 (1.02)

(ES)  =  effect size; TTPO  =  Time-trial mean power output; TT time  =  performance time; PPO  =  peak power output; VO_2_ peak  =  peak oxygen uptake; OBLA  =  onset blood lactate accumulation; ECO  =  exercise economy; GE  =  gross efficiency.

Table 2 shows the relative change score (as a percentage) for all measured variables between the three groups. There were moderate to large (ES  = 0.57–0.89) gains in performance measures for both training groups relative to the control condition; however the magnitude of changes in performance measures between the two training conditions were all considered small (ES <0.50). Differences in the change scores for VO_2_ peak, power at the lactate threshold and aerobic economy between the two training groups were trivial, whilst the long HIT group experienced a small (ES  = 0.34) increase in gross efficiency relative to the short HIT group.

**Table 2:** Pairwise comparison of changes in performance and physiological measures between all experimental groups.

	Long Control % difference ±90% CL† (ES)	Short – Control % difference ±90% CL (ES)	Short – Long % difference ±90% CL (ES)
**TTPO (W)**	10.4±4.3 (0.82)*	8.2±3.8 (0.67)#	−2.1±3.9 (−0.22)
**TT time(s)**	−6.1±2.2 (−0.80)#	−4.6±1.9 (−0.62)#	1.6±2.6 (0.28)
**PPO (W)**	9.5±2.5 (0.89)#	5.5±2.7 (0.57)#	−3.7±2.1 (−0.48)*
**VO_2_ peak (L·min^−1^)**	4.2±5.1 (0.37)	2.9±4.6 (0.27)	−1.2±4.2 (−0.15)
**OBLA (W)**	6.8±4.9 (0.53)*	7.5±4.5 (0.60)*	0.7±3.5 (0.05)
**ECO (W·L^−1^·min^−1^)**	2.3±3.2 (0.43)	1.7±3.0 (0.26)	−0.6±2.4 (−0.11)
**GE (%)**	3.6±3.3 (0.65)	1.7±2.9 (0.26)	−1.9±2.5 (−0.34)

† ±90% confidence limits: add or subtract this number to the mean effect to obtain the 90% confidence limits for the true difference. (ES)  =  effect size; TTPO  =  Time-trial mean power output; TT time  =  performance time; PPO  =  peak power output; VO_2_ peak  =  peak oxygen uptake; OBLA  =  onset blood lactate accumulation; ECO  =  exercise economy; GE  =  gross efficiency.

*significantly different at p<0.05.

# significantly different at p<0.01.

**Figure 3:**
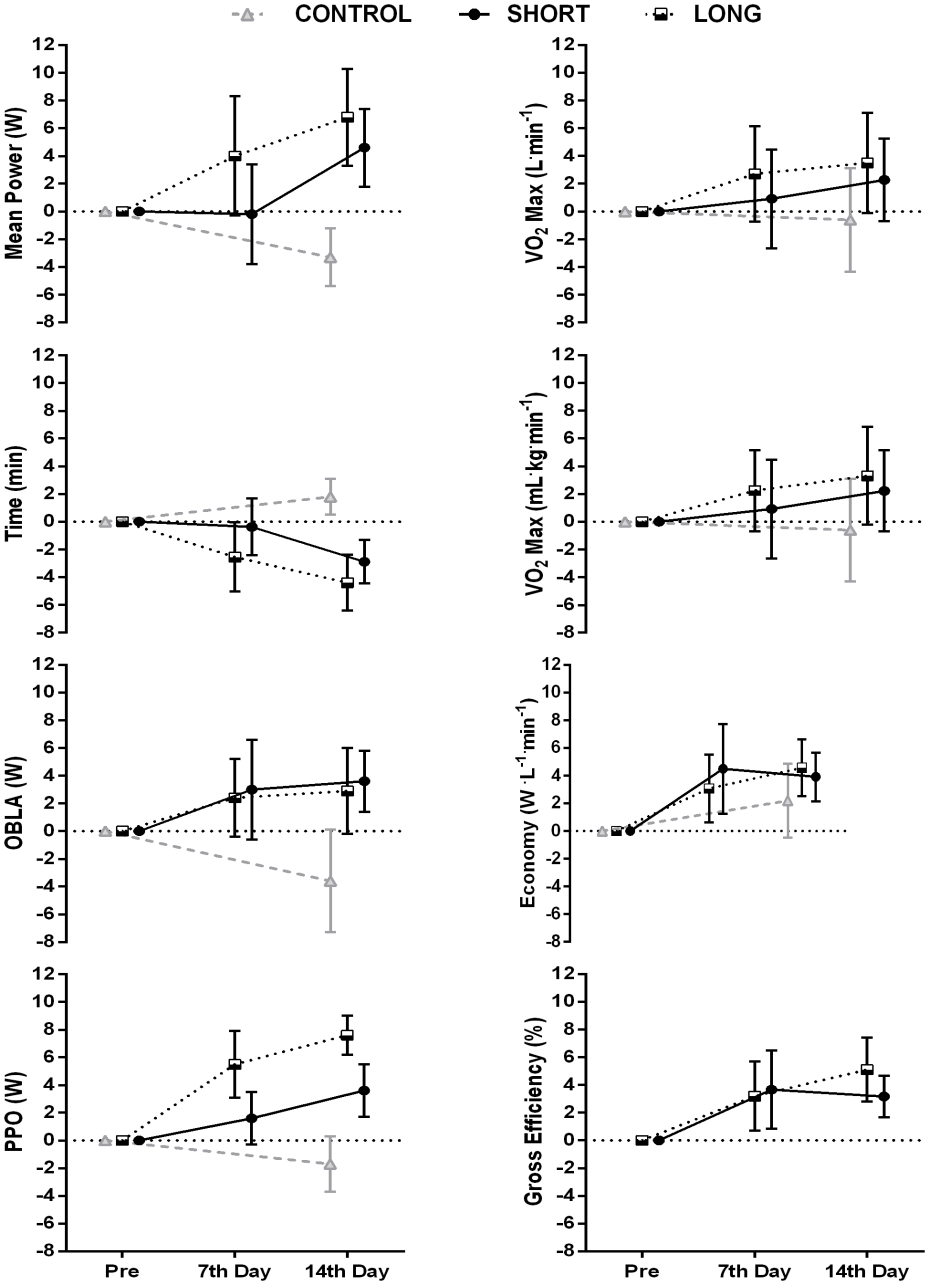
Shows the mean (±90% CL) percentage change in performance and physiological measures from baseline (pre) at the 7th day (post 1) and 14th day (post 2) after the HIT training period.

The mean (±90% confidence limits) percentage changes in performance and physiological measures (relative to baseline values) at both one week and two weeks post training is shown in Fig. 3. Whilst both experimental training groups experienced substantial gains in performance at the final post training (14 days) tests relative to the control group, the short HIT group experienced a delayed improvement in their actual time trial performance time and power in the first post training test (7 days) in comparison to the long HIT group.

Raw performance and physiological data for this study can be found in the provided supporting information: see S1 Data.

## Discussion

The aim of the present study was to examine the effects of 7-days of two pre-competition HIT regimes on the physiological and performance characteristics of competitive cyclists. Results from this study show that multiple sets of maximal short or long-duration efforts completed on consecutive days leads to substantial improvement in 20-km time trial performance in competitive cyclists. In addition whilst non-significant, both HIT regimes examined lead to enhancements in the key physiological determinates commonly associated with endurance performance.

Several previous authors have reported substantial performance gains following HIT in trained cyclists [3]–[7], [21]–[24]. In these studies HIT was associated with improvements in the main physiological variables associated with endurance performance, namely VO_2_ max [4], [6], [21], [24], [25], anaerobic threshold power [6], [24], aerobic economy [7] and gross efficiency [26]. However in all of these previous investigations, HIT was implemented over several weeks to months and typically in regimes that included just 2–3 interval training sessions per week. In comparison, cyclists in the current study experienced similar gains to those in previous studies after completing only seven consecutive days of HIT sessions. The findings in the current study therefore add empirical support to our observations that competitive athletes commonly use short blocks of intensified training to improve form prior to major competitions.

Similar rapid and substantial gains in performance and physiology have previously been reported with alpine skiers who performed 15 session of HIT over 11-days [27]. However a major difference between the current study and that of Breil et al., [27] is the latter study was completed with non-endurance trained individuals (alpine skiers) and in the athletes off season where there is much greater range for improvement due to their lower level of fitness. We also believe further evidence for the efficacy of short blocks of HIT comes from a more recent study by Ronnestad et al., [11]. While this previous study actually reported changes over a longer training period than the current study, they did include an initial 5-day intensified training block at the beginning of their 4-week training period. While it lacks specific performance testing following the initial HIT block, training data presented in these authors paper appears to indicate significant increases in training power output in the three weeks following the initial 5-days of training. We would therefore expect these increases in training power to also manifest as improvements in performance tests.

Whilst both the short and long HIT programmes in the current study led to substantial performance enhancements relative to the control group, the magnitude of change in performance measures between the two HIT programmes 2-wks post training were assessed as qualitatively small (ES∼0.2). Similarly differences in changes in physiological measures between the two HIT regimes were assessed as being trivial (ES <0.2) with the exception of gross efficiency (ES  = 0.34) which tended to a larger improvements in the long HIT group. Whilst the magnitude of performance difference between the two HIT strategies was small, this difference may be substantial enough to provide a worthwhile advantage during a real competition [19]. However while we tentatively suggest that there is a potentially greater improvements in the group performing the longer form of HIT, a study with a much larger sample size and clearer confidence limits would be required to verify this suggestion.

Further support for our opinion of the superiority of the long HIT form as the preferred training regime also comes from the differences in the rate of post training recovery in performance between the two HIT regimes. During the first post-training testing subjects in the short HIT showed no improvement in time trial performance relative to their pre-training test (Fig. 1) despite small improvements in performance measures during the short duration incremental test. We interpret this finding to indicate that the short HIT group had residual fatigue and insufficient recovery to gain any benefits from the training regime at this stage. Indeed, previous research examining the effects of short term overreaching has reported similar performance decrements in cyclists one week post a HIT intervention [13], [14]. A possible explanation for the performance difference between the two groups at this stage could relate to differences in the intensity of efforts in the training sessions. Whilst both groups were matched closely for total duration (volume) of both exercise and recovery, it is possible that the overall intensity of shorter sprints was somewhat higher than the longer efforts and therefore the short HIT is likely to have experienced greater cumulative fatigue. Indeed case study evaluations (unpublished observations) after the main study indicate that mean power in the short intervals was ∼10% higher than in the long intervals for the same total duration of effort. However we cannot exclude the possibility that the delayed improvement in the short HIT group is simply due to individual differences in the groups and sampling variation.

The contributions of physiological mediators underpinning the enhancement in time trial performance in both the HIT groups are unclear. While both HIT forms enhanced all measured physiological characteristics, the range of individual responses makes a precise determination of the contribution from any single mechanism difficult. Nevertheless an observational analysis of the improvements in the groups leads us to suggests that improvements in the long HIT group are more likely associated with increases in the cyclists aerobic economy (4.6%) and gross efficiency (5.1%) while improvement for the short HIT group appear associated with an increase in lactate threshold (OBLA) power (3.6%). Further it is possible other un-measured mechanism variable contributed to the performance enhancements. Indeed given the HIT interventions in the current study involved repeated maximal sprints, an increase in anaerobic and muscle buffering capacity could be expected as has been reported in previous studies examining the effects of HIT on time trial performance [6], [22]. However further investigations with a larger sample size, and additional measures related to biochemical adaptations [28] and mitochondrial biogenesis [29], would be necessary to further elucidate the potential molecular mechanisms responsible for any performance enhancements.

In conclusion one week of self-paced high-intensity overload training performed as multiple sets of short (5–20 s) or long (15–45 s) duration efforts led to substantial improvements in time-trial performance with competitive cyclist. The increases in performance were associated with enhancements in the three main mediators of endurance performance, VO_2_ peak, power output at the lactate threshold, and aerobic economy. While both long and short HIT sessions led to substantial increases in performance compared to the control group, the differences between the two training groups were generally small. However, although both the short and long interval programmes were closely matched for total exercise and recovery duration it appears the shorter intervals led to greater short-term decrements in time trial performance and required a longer post-training recovery period in order for any benefits to be realised. From a practical perspective we would recommend athletes planning to undertake block periods of intensified training, prior to competition, to opt for a combination of longer intervals or allow for a longer recovery prior to the competition if using shorter more intense intervals during their block training period. The findings of this study are of course limited to the training of competitive cyclists, but may be applicable to similar non-weight bearing aerobic sports (e.g. swimming and rowing). However caution is advised if trying to apply such an intense training routine to sports such as running, as the increased impact may lead to a greater injury risk and long term decline in performance. More studies are necessary with other sports to establish if these short intense periods of training are beneficial to performance. Further studies are also warranted to examine more closely the physiological mechanisms that underpin improvements in performance following intensified training and also to establish the time course over which any performance benefits are retained or lost.

## Supporting Information

S1 Data **Raw performance and physiological test data from the control, short interval, and long interval training groups.**
(PDF)
