# Supplementary material for: Effects of a Seven Day Overload-Period of High-Intensity Training on Performance and Physiology of Competitive Cyclists
Source: PLoS One. 2014 Dec 18;9(12):e115308. doi: 10.1371/journal.pone.0115308 (PMC4270748; doi:10.1371/journal.pone.0115308)
Supplement: S1 Data — Raw performance and physiological test data from the control, short interval, and long interval training groups. (PDF) [file pone.0115308.s001.pdf]

CONTROL GROUP TEST 1

| Name | AGE | WEIGHT | COMP WL | TIME FWL | PPO | PPO/KG | VT | VT/KG | VT HR | (OBLA) | LIP/KG | LIP HR | TT PO | TT TIME | TT HR | TT RPM | EC AT 220W | EFF | ABS VO2 | PEAK VE |
|---|---|---|---|---|---|---|---|---|---|---|---|---|---|---|---|---|---|---|---|---|
| GS | 36 | 84 | 380 | 15 | 383 | 4.6 | 308 | 3.7 | 156 | 325 | 3.9 | 161 | 297 | 2161 | 159 | 83 | 71.1 | 20 | 5.6617 | 174 |
| BT | 38 | 82 | 380 | 15 | 383 | 4.7 | 315 | 3.8 | 170 | 305 | 3.7 | 170 | 278 | 2356 | 164 | 88 | 76.5 | 22 | 5.093 | 192 |
| JS | 41 | 78 | 300 | 75 | 313 | 4.0 | 260 | 3.3 | 165 | 270 | 3.5 | 169 | 261 | 2375 | 175 | 94 | 70.3 | 21 | 4.5628 | 160 |
| LO | 28 | 78.3 | 420 | 0 | 420 | 5.4 | 320 | 4.1 | 150 | 355 | 4.5 | 160 | 347 | 2073 | 155 | 88 | 69.5 | 21 | 5.753 | 168 |
| MK | 42 | 73 | 300 | 135 | 323 | 4.4 | 248 | 3.4 | 158 | 258 | 3.5 | 163 | 263 | 2369 | 170 | 100 | 69.0 | 20 | 4.323 | 160 |
| BC | 25 | 63 | 340 | 17 | 343 | 5.4 | 300 | 4.8 | 181 | 282 | 4.5 | 174 | 305 | 2129 | 185 | 88 | 80.7 | 23 | 4.27 | 144.6 |
| MC | 21 | 64 | 380 | 69 | 392 | 6.1 | 353 | 5.5 | 168 | 356 | 5.6 | 166 | 365 | 1959 | 175 | 88 | 74.8 | 22 | 4.95 | 150.9 |
| PC | 48 | 73 | 300 | 188 | 331 | 4.5 | 273 | 3.7 | 161 | 285 | 3.9 | 164 | 280 | 2284 | 176 | 82 | 69.4 | 20 | 4.56 | 158.4 |
| SS | 47 | 80 | 340 | 80 | 353 | 4.4 | 300 | 3.8 | 174 | 301 | 3.8 | 174 | 297 | 2281 | 175 | 84 | 70.7 | 21 | 4.36 | 144.8 |
| TD | 37 | 91 | 260 | 142 | 284 | 3.1 | 238 | 2.6 | 155 | 247 | 2.7 | 158 | 228 | 2697 | 156 | 84 | 69.8 | 20 | 3.74 | 138.8 |
| **AVE** | **36.3** | **76.6** | **340** | **74** | **352** | **4.7** | **292** | **3.9** | **164** | **298** | **4.0** | **166** | **292** | **2268** | **169** | **88** | **72** | **21.0** | **4.73** | **159** |
| **SD** | **9.1** | **8.7** | **49.9** | **64.2** | **41.7** | **0.8** | **36.0** | **0.8** | **9.6** | **37.7** | **0.8** | **5.7** | **40.6** | **205.0** | **10.0** | **5.5** | **3.9** | **1.1** | **0.6** | **15.9** |

CONTROL GROUP TEST 2

| NAME | AGE | WEIGHT | COMP WL | TIME FWL | PPO | PPO/KG | VT | VT/KG | VT HR | (OBLA) | LIP/KG | LIP HR | TT PO | TT TIME | TT HR | TT RPM | EC AT 220W | EFF | ABS VO2 | PEAK VE |
|---|---|---|---|---|---|---|---|---|---|---|---|---|---|---|---|---|---|---|---|---|
| GS | 36 | 84 | 380 | 30 | 385 | 4.6 | 290 | 3.5 | 151 | 323 | 3.8 | 160 | 303 | 2139 | 157 | 79 | 78 | 22.4 | 5.159 | 162 |
| BT | 38 | 82 | 340 | 205 | 374 | 4.6 | 320 | 3.9 | 165 | 309 | 3.8 | 161 | 277 | 2389 | 157 | 79 | 78 | 22.4 | 5.357 | 175.4 |
| JS | 41 | 78 | 300 | 30 | 305 | 3.9 | 253 | 3.2 | 156 | 288 | 3.7 | 166 | 232 | 2533 | 152 | 89 | 72 | 20.6 | 4.2416 | 150 |
| LO | 28 | 78.3 | 380 | 90 | 395 | 5.0 | 305 | 3.9 | 144 | 340 | 4.3 | 154 | 304 | 2203 | 146 | 94 | 63 | 18.2 | 5.808 | 154.1 |
| MK | 42 | 73 | 300 | 90 | 315 | 4.3 | 250 | 3.4 | 157 | 250 | 3.4 | 157 | 256 | 2403 | 167 | 100 | 72 | 20.7 | 4.323 | 166 |
| BC | 25 | 63 | 300 | 120 | 320 | 5.1 | 293 | 4.7 | 183 | 249 | 4.0 | 173 | 286 | 2198 | 184 | 85 | 79 | 22.6 | 4.25 | 142.1 |
| MC | 21 | 64 | 380 | 63 | 391 | 6.1 | 340 | 5.3 | 169 | 331 | 5.2 | 166 | 357 | 1981 | 173 | 88 | 78 | 23.2 | 5.08 | 167.8 |
| PC | 48 | 73 | 300 | 75 | 313 | 4.3 | 273 | 3.7 | 161 | 256 | 3.5 | 156 | 275 | 2309 | 175 | 81 | 73 | 20.9 | 4.26 | 148.5 |
| SS | 47 | 80 | 340 | 80 | 353 | 4.4 | 300 | 3.8 | 169 | 303 | 3.8 | 169 | 280 | 2343 | 171 | 84 | 71 | 20.7 | 4.45 | 140.5 |
| TD | 37 | 91 | 260 | 205 | 294 | 3.2 | 233 | 2.6 | 162 | 230 | 2.5 | 162 | 226 | 2700 | 159 | 85 | 66 | 19.0 | 4.08 | 143.6 |
| **AVE** | **36.3** | **76.6** | **328** | **99** | **344** | **4.6** | **286** | **3.8** | **162** | **288** | **3.8** | **162** | **280** | **2320** | **164** | **86** | **73** | **21.1** | **4.70** | **155** |
| **SD** | **9.1** | **8.7** | **42.4** | **62.2** | **39.2** | **0.8** | **33.4** | **0.8** | **10.8** | **39.2** | **0.7** | **6.1** | **37.9** | **205.0** | **11.8** | **6.7** | **5.4** | **1.6** | **0.6** | **12.1** |

CRASH SHORT TEST 1

| NAME | AGE | WEIGHT | COMP WL | TIME FWL | PPO | PPO/KG | VT | VT/KG | VT HR | (OBLA) | LIP/KG | LIP HR | TT PO | TT TIME | TT HR | TT RPM | EC AT 220W | EFF | ABS VO2 | PEAK VE |
|---|---|---|---|---|---|---|---|---|---|---|---|---|---|---|---|---|---|---|---|---|
| AS | 38 | 68 | 300 | 191 | 332 | 4.9 | 260 | 3.8 | 153 | 236 | 3.5 | 147 | 254 | 2383 | 157 | 88 | 69 | 19.9 | 4.479 | 152.3 |
| BC | 25 | 62 | 340 | 21 | 344 | 5.5 | 280 | 4.5 | 170 | 284 | 4.6 | 171 | 312 | 2087 | 185 | 93 | 72.2 | 21 | 4.884 | 140.5 |
| CS | 39 | 79 | 300 | 219 | 337 | 4.3 | 260 | 3.3 | 151 | 200 | 2.5 | 129 | 278 | 2327 | 170 | 87 | 63.2 | 18 | 5.22 | 193.2 |
| JL | 17 | 67 | 300 | 109 | 318 | 4.7 | 263 | 3.9 | 174 | 274 | 4.1 | 178 | 231 | 2548 | 174 | 84 | 70.1 | 20 | 4.371 | 133.5 |
| MT | 35 | 72 | 340 | 0 | 340 | 4.7 | 255 | 3.5 | 151 | 297 | 4.1 | 165 | 257 | 2374 | 157 | 98 | 58.9 | 18 | 4.646 | 192.9 |
| PC | 48 | 71 | 300 | 94 | 316 | 4.4 | 258 | 3.6 | 167 | 277 | 3.9 | 172 | 265 | 2342 | 176 | 88 | 72.2 | 21 | 4.467 | 164.4 |
| RH | 23 | 77 | 300 | 89 | 315 | 4.1 | 263 | 3.4 | 153 | 274 | 3.6 | 156 | 267 | 2364 | 168 | 89 | 68.2 | 20 | 4.685 | 163.6 |
| RK | 35 | 82 | 380 | 0 | 380 | 4.6 | 305 | 3.7 | 143 | 270 | 3.3 | 132 | 300 | 2307 | 158 | 104 | 75.7 | 22 | 5.219 | 185.1 |
| TB | 17 | 69 | 340 | 147 | 365 | 5.3 | 300 | 4.3 | 177 | 284 | 4.1 | 172 | 298 | 2200 | 176 | 83 | 76.9 | 22 | 4.686 | 176.9 |
| **AVE** | **30.8** | **71.9** | **322.2** | **96.7** | **338.3** | **4.7** | **271.6** | **3.8** | **159.9** | **266.2** | **3.7** | **158.0** | **273.6** | **2325.8** | **169.0** | **90.4** | **69.6** | **20.3** | **4.7** | **166.9** |
| **SD** | **10.8** | **6.4** | **29.1** | **79.9** | **22.3** | **0.5** | **18.9** | **0.4** | **12.2** | **29.9** | **0.6** | **18.2** | **25.9** | **127.4** | **9.9** | **6.8** | **5.7** | **1.5** | **0.3** | **21.9** |

CRASH SHORT TEST 2

| NAME | AGE | WEIGHT | COMP WL | TIME FWL | PPO | PPO/KG | VT | VT/KG | VT HR | (OBLA) | LIP/KG | LIP HR | TT PO | TT TIME | TT HR | TT RPM | EC AT 220W | EFF | ABS VO2 | PEAK VE |
|---|---|---|---|---|---|---|---|---|---|---|---|---|---|---|---|---|---|---|---|---|
| AS | 38 | 68 | 340 | 20 | 343 | 5.0 | 265 | 3.9 | 153 | 234 | 3.4 | 143 | 267 | 2318 | 156 | 87 | 70.2 | 21 | 4.542 | 154.9 |
| BC | 25 | 62 | 340 | 116 | 359 | 5.8 | 295 | 4.8 | 173 | 272 | 4.4 | 168 | 319 | 2067 | 182 | 88 | 75.0 | 22 | 4.708 | 167.3 |
| CS | 39 | 79 | 340 | 44 | 347 | 4.4 | 260 | 3.3 | 142 | 248 | 3.1 | 136 | 299 | 2244 | 164 | 85 | 68.0 | 20 | 4.887 | 201.1 |
| JL | 17 | 67 | 300 | 180 | 330 | 4.9 | 265 | 4.0 | 179 | 266 | 4.0 | 179 | 267 | 2306 | 182 | 84 | 74.2 | 21 | 4.166 | 149.5 |
| MT | 35 | 72 | 300 | 178 | 330 | 4.6 | 263 | 3.7 | 152 | 273 | 3.8 | 155 | 262 | 2368 | 156 | 94 | 72.1 | 21 | 4.607 | 179.5 |
| PC | 48 | 71 | 300 | 105 | 318 | 4.5 | 260 | 3.7 | 156 | 271 | 3.8 | 168 | 270 | 2318 | 172 | 88 | 71.0 | 21 | 4.376 | 149.3 |
| RH | 23 | 77 | 300 | 163 | 327 | 4.2 | 260 | 3.4 | 151 | 260 | 3.4 | 152 | 267 | 2364 | 168 | 89 | 69.5 | 20 | 4.815 | 170.9 |
| RK | 35 | 82 | 380 | 46 | 388 | 4.7 | 305 | 3.7 | 142 | 281 | 3.4 | 134 | 319 | 2229 | 156 | 94 | 75.5 | 22 | 5.335 | 204.3 |
| TB | 17 | 69 | 340 | 81 | 354 | 5.1 | 300 | 4.3 | 175 | 280 | 4.1 | 169 | 288 | 2238 | 175 | 79 | 81.3 | 23 | 4.303 | 154.8 |
| **AVE** | **30.8** | **71.9** | **326.7** | **103.7** | **343.9** | **4.8** | **274.8** | **3.9** | **158.1** | **265.0** | **3.7** | **156.0** | **284.2** | **2272.4** | **167.9** | **87.6** | **73.0** | **21.1** | **4.6** | **170.2** |
| **SD** | **10.8** | **6.4** | **28.3** | **60.6** | **21.3** | **0.5** | **19.2** | **0.5** | **14.1** | **15.4** | **0.4** | **16.0** | **23.0** | **92.8** | **10.6** | **4.7** | **4.1** | **1.1** | **0.4** | **21.1** |

## CRASH SHORT TEST 3

| NAME | AGE | WEIGHT | COMP WL | TIME FWL | PPO | PPO/KG | VT | VT/KG | VT HR | (OBLA) | LIP/KG | LIP HR | TT PO | TT TIME | TT HR | TT RPM | EC AT 220W | EFF | ABS V02 | PEAK VE |
|---|---|---|---|---|---|---|---|---|---|---|---|---|---|---|---|---|---|---|---|---|
| AS | 38 | 68 | 340 | 30 | 345 | 5.1 | 260 | 3.8 | 151 | 264 | 3.9 | 152 | 267 | 2295 | 155 | 87 | 71.7 | 21 | 4.796 | 166.6 |
| BC | 25 | 62 | 340 | 52 | 349 | 5.6 | 290 | 4.7 | 172 | 279 | 4.5 | 168 | 315 | 2079 | 187 | 84 | 74.8 | 21 | 4.526 | 150.4 |
| CS | 39 | 79 | 300 | 191 | 332 | 4.2 | 260 | 3.3 | | 255 | 3.2 | | 265 | 2369 | | 80 | 71.3 | 21 | 4.732 | 204.3 |
| JL | 17 | 67 | 340 | 30 | 345 | 5.1 | 263 | 3.9 | 168 | 273 | 4.1 | 172 | 282 | 2239 | 179 | 82 | 74.8 | 22 | 4.442 | 155.8 |
| MT | 35 | 72 | 300 | 225 | 338 | 4.7 | 263 | 3.7 | 152 | 296 | 4.1 | 162 | 258 | 2376 | 156 | 90 | 76.8 | 22 | 4.797 | 177.1 |
| PC | 48 | 71 | 300 | 148 | 325 | 4.6 | 260 | 3.7 | 164 | 267 | 3.8 | 166 | 256 | 2368 | 178 | 81 | 75.1 | 22 | 4.221 | 162.8 |
| RH | 23 | 77 | 340 | 1 | 340 | 4.4 | 263 | 3.4 | | 263 | 3.4 | | 267 | 2372 | 156 | 87 | 68.7 | 20 | 5.195 | 188.2 |
| RK | 35 | 82 | 380 | 52 | 389 | 4.7 | 300 | 3.7 | 143 | 284 | 3.5 | 138 | 310 | 2258 | 157 | 90 | 77.2 | 23 | 5.507 | 210.7 |
| TB | 17 | 69 | 340 | 90 | 355 | 5.1 | 303 | 4.4 | 177 | 277 | 4.0 | 167 | 283 | 2255 | 175 | 82 | 79.3 | 23 | 4.417 | 162.7 |
| **AVE** | **30.8** | **71.9** | **331.1** | **91.0** | **346.3** | **4.8** | **273.6** | **3.8** | **161.0** | **273.1** | **3.8** | **160.7** | **278.1** | **2290.1** | **167.9** | **84.8** | **74.4** | **21.4** | **4.7** | **175.4** |
| **SD** | **10.8** | **6.4** | **26.7** | **78.9** | **18.3** | **0.4** | **18.4** | **0.4** | **12.5** | **12.5** | **0.4** | **11.8** | **21.6** | **97.4** | **13.1** | **3.8** | **3.3** | **1.0** | **0.4** | **21.4** |

## CRASH SHORT TEST 4

| NAME | AGE | WEIGHT | COMP WL | TIME FWL | PPO | PPO/KG | VT | VT/KG | VT HR | (OBLA) | LIP/KG | LIP HR | TT PO | TT TIME | TT HR | TT RPM | EC AT 220W | EFF | ABS V02 | PEAK VE |
|---|---|---|---|---|---|---|---|---|---|---|---|---|---|---|---|---|---|---|---|---|
| AS | 38 | 68 | 340 | 100 | 357 | 5.2 | 270 | 4.0 | 151 | 244 | 3.6 | 143 | 272 | 2275 | 154 | 84 | 74.0 | 21 | 4.534 | 170.9 |
| BC | 25 | 62 | 340 | 80 | 353 | 5.7 | 290 | 4.7 | 171 | 295 | 4.8 | 172 | 322 | 2055 | 187 | 82 | 74.8 | 22 | 4.609 | 153.4 |
| CS | 39 | 79 | 300 | 215 | 336 | 4.3 | 260 | 3.3 | 143 | 221 | 2.8 | 137 | 280 | 2323 | 165 | 79 | 67.4 | 19 | 4.774 | 202.6 |
| JL | 17 | 67 | 340 | 61 | 350 | 5.2 | 273 | 4.1 | 171 | 278 | 4.1 | 172 | 281 | 2242 | 179 | 80 | 76.4 | 22 | 4.407 | 152.3 |
| MT | 35 | 72 | 340 | 30 | 345 | 4.8 | 260 | 3.6 | 153 | 296 | 4.1 | 164 | 272 | 2314 | 159 | 95 | 70.0 | 20 | 4.844 | 182.8 |
| PC | 48 | 71 | 300 | 200 | 333 | 4.7 | 283 | 4.0 | 161 | 283 | 4.0 | 161 | 291 | 2225 | 178 | 81 | 75.5 | 22 | 4.721 | 159.2 |
| RH | 23 | 77 | 340 | 11 | 342 | 4.4 | 260 | 3.4 | 3.72 | 265 | 3.4 | 157 | 283 | 2278 | 165 | 77 | 71.8 | 21 | 5.067 | 174.7 |
| RK | 35 | 82 | 380 | 81 | 394 | 4.8 | 300 | 3.7 | 143 | 306 | 3.7 | 145 | 313 | 2223 | 153 | 90 | 78.3 | 23 | 5.69 | 205.2 |
| TB | 17 | 69 | 340 | 184 | 371 | 5.4 | 308 | 4.5 | 173 | 293 | 4.2 | 169 | 308 | 2154 | 177 | 85 | 77.6 | 22 | 4.545 | 174.4 |
| **AVE** | **30.8** | **71.9** | **335.6** | **106.9** | **353.4** | **4.9** | **278.2** | **3.9** | **141.1** | **275.7** | **3.9** | **157.8** | **291.3** | **2232.1** | **168.6** | **83.7** | **74.0** | **21.3** | **4.8** | **175.1** |
| **SD** | **10.8** | **6.4** | **24.0** | **75.0** | **18.9** | **0.5** | **18.1** | **0.5** | **52.8** | **27.8** | **0.6** | **13.2** | **18.5** | **84.1** | **12.1** | **5.7** | **3.6** | **1.0** | **0.4** | **19.3** |

CRASH LONG TEST 1

| NAME | AGE | WEIGHT | COMP WL | TIME FWL | PPO | PPO/KG | VT | VT/KG | VT HR | (OBLA) | LIP/KG | LIP HR | TT PO | TT TIME | TT HR | TT RPM | EC AT 220W | EFF | ABS VO2 | PEAK VE |
|---|---|---|---|---|---|---|---|---|---|---|---|---|---|---|---|---|---|---|---|---|
| BR | 16 | 78.7 | 340 | 165 | 368 | 4.7 | 320 | 4.1 | 171 | 302 | 3.8 | 165 | 298 | 2246 | 174 | 103 | 73 | 20.8 | 4.664 | 162 |
| CP | 45 | 70 | 300 | 60 | 310 | 4.4 | 248 | 3.5 | 165 | 245 | 3.5 | 165 | 251 | 2421 | 171 | 82 | 75 | 21.2 | 3.905 | 175 |
| GH | 37 | 72.8 | 340 | 165 | 368 | 5.0 | 300 | 4.1 | 146 | 329 | 4.5 | 157 | 289 | 2263 | 159 | 99 | 67 | 19.5 | 5.4109 | 180 |
| JK | 42 | 71.7 | 340 | 75 | 353 | 4.9 | 263 | 3.7 | 131 | 331 | 4.6 | 128 | 298 | 2235 | 154 | 93 | 72 | 20.9 | 4.6145 | 179 |
| KW | 44 | 68 | 300 | 45 | 308 | 4.5 | 253 | 3.7 | 156 | 270 | 4.0 | 164 | 254 | 2387 | 164 | 90 | 77 | 22.0 | 4.0623 | 175 |
| NIGE | 42 | 86 | 340 | 90 | 355 | 4.1 | 300 | 3.5 | 162 | 331 | 3.8 | 171 | 297 | 2181 | 172 | 90 | 69 | 19.8 | 5.005 | 185 |
| NIK | 30 | 83 | 300 | 30 | 305 | 3.7 | 250 | 3.0 | 165 | 254 | 3.1 | 168 | 235 | 2670 | 171 | 114 | 67 | 19.6 | 5.038 | 165 |
| VC | 33 | 75 | 300 | 0 | 300 | 4.0 | 255 | 3.4 | 177 | 249 | 3.3 | 175 | 248 | 2440 | 178 | 92 | 67 | 19.1 | 4.367 | 165 |
| WB | 27 | 74.9 | 340 | 105 | 358 | 4.8 | 308 | 4.1 | 151 | 327 | 0.0 | 156 | 315 | 2153 | 159 | 89 | 70 | 20.8 | 4.994 | 157 |
| WG | 16 | 67.8 | 300 | 150 | 325 | 4.8 | 275 | 4.1 | 170 | 278 | 4.1 | 170 | 274 | 2337 | 173 | 93 | 71 | 20.2 | 4.389 | 170 |
| **AVE** | **33.2** | **74.8** | **320.0** | **88.5** | **334.8** | **4.5** | **277.2** | **3.7** | **159.4** | **291.6** | **3.5** | **161.9** | **275.9** | **2333.3** | **167.5** | **94.5** | **70.8** | **20.4** | **4.6** | **171.3** |
| **SD** | **10.9** | **6.1** | **21.1** | **57.6** | **27.8** | **0.4** | **27.3** | **0.4** | **13.7** | **36.4** | **1.3** | **13.3** | **27.2** | **153.9** | **7.9** | **8.9** | **3.4** | **0.9** | **0.5** | **9.0** |

CRASH LONGTEST 2

| NAME | AGE | WEIGHT | COMP WL | TIME FWL | PPO | PPO/KG | VT | VT/KG | VT HR | (OBLA) | LIP/KG | LIP HR | TT PO | TT TIME | TT HR | TT RPM | EC AT 220W | EFF | ABS VO2 | PEAK VE |
|---|---|---|---|---|---|---|---|---|---|---|---|---|---|---|---|---|---|---|---|---|
| BR | 16 | 78.7 | 340 | 15 | 343 | 4.4 | 303 | 3.9 | 164 | 321 | 4.1 | 169 | 261 | 2427 | 158 | 96 | 76 | 21.8 | 5.181 | 142 |
| CP | 45 | 70 | 300 | 105 | 318 | 4.5 | 263 | 3.8 | 170 | 270 | 3.9 | 172 | 260 | 2366 | 172 | 83 | 74 | 21.0 | 4.268 | 177 |
| GH | 37 | 72.8 | 340 | 225 | 378 | 5.2 | 310 | 4.3 | 144 | 318 | 4.4 | 147 | 300 | 2189 | 158 | 97 | 78 | 22.5 | 5.1634 | 172 |
| JK | 42 | 71.7 | 340 | 105 | 358 | 5.0 | 278 | 3.9 | 126 | 328 | 4.6 | 145 | 311 | 2178 | 150 | 92 | 75 | 21.7 | 4.818 | 197 |
| KW | 44 | 68 | 300 | 15 | 303 | 4.4 | 258 | 3.8 | 169 | 272 | 4.0 | 162 | 260 | 2360 | 165 | 81 | 76 | 21.8 | 4.3714 | 174 |
| NIGE | 42 | 86 | 340 | 105 | 358 | 4.2 | 300 | 3.5 | 159 | 356 | 4.1 | 173 | 293 | 2202 | 162 | 92 | 72 | 20.9 | 4.631 | 185 |
| NIK | 30 | 83 | 300 | 90 | 315 | 3.8 | 260 | 3.1 | 168 | 299 | 3.6 | 177 | 255 | 2497 | 177 | 92 | 68 | 19.9 | 4.708 | 155 |
| VC | 33 | 75 | 300 | 15 | 303 | 4.0 | 260 | 3.5 | 176 | 247 | 3.3 | 172 | 244 | 2449 | 178 | 90 | 64 | 18.5 | 4.367 | 172 |
| WB | 27 | 74.9 | 340 | 205 | 374 | 5.0 | 300 | 4.0 | 148 | 332 | 4.4 | 159 | 323 | 2126 | 165 | 87 | 72 | 21.2 | 5.093 | 173 |
| WG | 16 | 67.8 | 340 | 0 | 340 | 5.0 | 285 | 4.2 | 163 | 291 | 4.3 | 165 | 279 | 2271 | 167 | 95 | 77 | 21.8 | 4.554 | 170 |
| **AVE** | **33.2** | **74.8** | **324.0** | **88.0** | **338.7** | **4.6** | **281.7** | **3.8** | **158.7** | **303.4** | **4.1** | **164.1** | **278.6** | **2306.5** | **165.2** | **90.5** | **73.2** | **21.1** | **4.7** | **171.7** |
| **SD** | **10.9** | **6.1** | **20.7** | **79.6** | **28.1** | **0.5** | **20.6** | **0.3** | **15.2** | **33.6** | **0.4** | **11.0** | **26.8** | **130.2** | **8.8** | **5.4** | **4.3** | **1.2** | **0.3** | **15.0** |

CRASH LONG TEST 3

| NAME | AGE | WEIGHT | COMP WL | TIME FWL | PPO | PPO/KG | VT | VT/KG | VT HR | (OBLA) | LIP/KG | LIP HR | TT PO | TT TIME | TT HR | TT RPM | EC AT 220W | EFF | ABS V02 | PEAK VE |
|---|---|---|---|---|---|---|---|---|---|---|---|---|---|---|---|---|---|---|---|---|
| BR | 16 | 78.7 | 340 | 225 | 378 | 4.8 | 315 | 4.0 | 166 | 322 | 4.1 | 170 | 295 | 2265 | 171 | 98 | 75 | 21.4 | 4.95 | 178 |
| CP | 45 | 71 | 340 | 30 | 345 | 4.9 | 263 | 3.7 | 164 | 281 | 4.0 | 168 | 292 | 2214 | 173 | 85 | 76 | 21.7 | 4.5441 | 176 |
| GH | 37 | 71.8 | 380 | 120 | 400 | 5.6 | 310 | 4.3 | 147 | 325 | 4.5 | 154 | 324 | 2087 | 165 | 90 | 76 | 22.2 | 5.522 | 183 |
| JK | 42 | 72.9 | 380 | 15 | 383 | 5.2 | 303 | 4.2 | 131 | 323 | 4.4 | 140 | 295 | 2261 | 148 | 96 | 72 | 21.0 | 4.9247 | 198 |
| KW | 44 | 68 | 300 | 30 | 305 | 4.5 | 260 | 3.8 | 161 | 272 | 4.0 | 166 | 248 | 2409 | 167 | 86 | 74 | 21.1 | 4.1503 | 189 |
| NIGE | 42 | 86.4 | 340 | 180 | 370 | 4.3 | 313 | 3.6 | 163 | 325 | 3.8 | 168 | 295 | 2188 | 176 | 88 | 72 | 20.9 | 5.214 | 193 |
| NIK | 30 | 82 | 340 | 0 | 340 | 4.1 | 285 | 3.5 | 168 | 296 | 3.6 | 170 | 276 | 2356 | 174 | 86 | 70 | 20.3 | 4.708 | 157 |
| VC | 33 | 75.2 | 300 | 120 | 320 | 4.3 | 268 | 3.6 | 183 | 257 | 3.4 | 179 | 264 | 2365 | 187 | 86 | 70 | 20.1 | 4.389 | 182 |
| WB | 27 | 74.9 | 380 | 75 | 393 | 5.2 | 350 | 4.7 | 159 | 362 | 4.8 | 162 | 338 | 2067 | 164 | 86 | 74 | 21.9 | 5.379 | 173 |
| WG | 16 | 67.8 | 300 | 145 | 324 | 4.8 | 260 | 3.8 | 167 | 283 | 4.2 | 170 | 257 | 2392 | 169 | 92 | 83 | 23.5 | 4.202 | 168 |
| **AVE** | **33.2** | **74.9** | **340.0** | **94.0** | **355.7** | **4.8** | **292.7** | **3.9** | **160.9** | **304.6** | **4.1** | **164.7** | **288.4** | **2260.4** | **169.4** | **89.3** | **74.2** | **21.4** | **4.8** | **179.7** |
| **SD** | **10.9** | **6.0** | **32.7** | **76.1** | **33.2** | **0.5** | **30.3** | **0.4** | **13.8** | **32.0** | **0.4** | **10.8** | **28.4** | **122.2** | **10.0** | **4.6** | **3.8** | **1.0** | **0.5** | **12.1** |

CRASH LONG TEST 4

| NAME | AGE | WEIGHT | COMP WL | TIME FWL | PPO | PPO/KG | VT | VT/KG | VT HR | (OBLA) | LIP/KG | LIP HR | TT PO | TT TIME | TT HR | TT RPM | EC AT 220W | EFF | ABS V02 | PEAK VE |
|---|---|---|---|---|---|---|---|---|---|---|---|---|---|---|---|---|---|---|---|---|
| BR | 16 | 78.9 | 380 | 45 | 388 | 4.9 | 325 | 4.1 | 167 | 302 | 3.8 | 171 | 316 | 2144 | 175 | 98 | 77 | 22.0 | 5.368 | 180 |
| CP | 45 | 71.4 | 340 | 15 | 343 | 4.8 | 283 | 4.0 | 163 | 275 | 3.9 | 163 | 290 | 2203 | 168 | 83 | 79 | 23.0 | 4.2185 | 179 |
| GH | 37 | 71.5 | 380 | 75 | 393 | 5.5 | 325 | 4.5 | 150 | 334 | 4.7 | 155 | 332 | 2069 | 165 | 92 | 80 | 23.1 | 5.291 | 171 |
| JK | 42 | 72 | 380 | 15 | 383 | 5.3 | 320 | 4.4 | 139 | 327 | 4.5 | 142 | 307 | 2190 | 150 | 86 | 72 | 20.9 | 5.3284 | 191 |
| KW | 44 | 68 | 300 | 120 | 320 | 4.7 | 255 | 3.8 | 154 | 266 | 3.9 | 156 | 263 | 2334 | 164 | 84 | 78 | 22.1 | 4.3593 | 192 |
| NIGE | 42 | 87 | 380 | 15 | 383 | 4.4 | 308 | 3.5 | 168 | 333 | 3.8 | 176 | 285 | 2222 | 172 | 83 | 71 | 20.7 | 5.137 | 196 |
| NIK | 30 | 82.7 | 340 | 15 | 343 | 4.1 | 283 | 3.4 | 166 | 312 | 3.8 | 174 | 274 | 2370 | 175 | 88 | 73 | 21.6 | 4.389 | 160 |
| VC | 33 | 74 | 300 | 210 | 335 | 4.5 | 263 | 3.6 | 174 | 255 | 3.4 | 172 | 266 | 2344 | 183 | 83 | 69 | 19.8 | 4.62 | 199 |
| WB | 27 | 76 | 380 | 75 | 393 | 5.2 | 350 | 4.6 | 159 | 362 | 4.8 | 162 | 330 | 2099 | 157 | 81 | 76 | 22.6 | 5.137 | 175 |
| WG | 16 | 68 | 340 | 30 | 345 | 5.1 | 300 | 4.4 | 169 | 297 | 4.4 | 169 | 296 | 2188 | 171 | 91 | 78 | 22.4 | 4.642 | 157 |
| **AVE** | **33.2** | **75.0** | **352.0** | **61.5** | **362.3** | **4.9** | **301.2** | **4.0** | **160.9** | **306.3** | **4.1** | **164.0** | **295.9** | **2216.3** | **168.0** | **86.9** | **75.3** | **21.8** | **4.8** | **180.0** |
| **SD** | **10.9** | **6.3** | **32.9** | **63.0** | **27.7** | **0.4** | **30.1** | **0.5** | **10.6** | **34.0** | **0.4** | **10.6** | **25.0** | **103.4** | **9.5** | **5.3** | **3.9** | **1.1** | **0.4** | **14.6** |
